# Post-traumatic stress disorder and association with low birth weight in displaced population following conflict in Malakand division, Pakistan: a case control study

**DOI:** 10.1186/s12884-020-2841-2

**Published:** 2020-03-17

**Authors:** Haroon Ur Rashid, Muhammad Naseem Khan, Ayesha Imtiaz, Naeem Ullah, Mukesh Dherani, Atif Rahman

**Affiliations:** 1 Institute of Public Health & Social Sciences (IPH&SS), Khyber Medical University, Phase V, Hayatabad, Peshawar, Pakistan; 2 Department of Psychological Medicine, University of Liverpool, Liverpool, UK; 3 Saidu Medical College, Swat, Pakistan; 4 Human Development Research Foundation, Islamabad, Pakistan

**Keywords:** Low birth weight, Post traumatic stress disorder, Pakistan, Conflict, Pregnancy outcome

## Abstract

**Background:**

The northern part of the province of Khyber Pakhtunkhwa in Pakistan experienced armed conflict since September 2007 till the autumn of 2011. Conflict involved widespread insurgency activity and military intervention including in 2009 internally displacing the 2.5 million people of the valley of Swat to live in camps, with relatives, or in rented accommodation across the region for approximately 4 months. It was during this period the current study was conducted to determine whether Post-Traumatic Stress Disorder in pregnant women was independently associated with Low Birth Weight (LBW) in an area affected by conflict and militancy.

**Methods:**

A case control study was conducted in tertiary care hospitals of district Peshawar, Khyber Pakhtunkhwa. Two hundred twenty-five cases (neonates with birth weight <  2.5 kg) and 225 controls (neonates with birth weight of > 2.5 kg) were enrolled within 24 h of delivery. Post-Traumatic Stress Disorder was assessed through the MINI Neuropsychiatric Interview 5.0, a validated questionnaire along with the birth weight of the newborn. Maternal anthropometry, anemia and other sociodemographic details were also obtained during data collection. Data was analyzed using statistical package (STATA version 14). Logistic regression analysis of the association between LBW and all variables collected with a *p*-value of < 0.25 on uni-variate analysis were entered.

**Results:**

A total of 450 newborn and mother pairs participated in the study with 225 cases and 225 controls. On univariate analysis factors significantly associated with LBW include: less than 5 years of paternal schooling and PTSD. On logistic regression, PTSD was independently associated with low birth weight in the presence of other factors like maternal/paternal schooling, gravida, history of preterm, BMI of the mother and maternal anemia.

**Conclusion:**

PTSD was found to be independently associated with LBW. In light of the current findings and other similar literature, intervention programs should be considered for pregnant women exposed to traumatic events.

## Background

There is a gradual rise in natural and man-made disasters in the past few decades, with a global estimates of the Internally Displaced Persons (IDPs) resulting from these conflicts since 1990 to 2009 exceeding 23 million. Alarmingly around half of these numbers have occurred in the Eastern Mediterranean Region (EMR) of the World Health Organization (WHO) which includes Pakistan [1, 2]. Majority of IDPs were exposed to dreadful conditions and violations of their basic human rights including the rights to health of mothers and children [2, 3]. The prevalence of common mental disorders in the conflict affected population was 22.1% [95% CI 18.8–25.7] while, that of Post-Traumatic Stress Disorder (PTSD) was 15.3% (95% CI [9.9–23.5] [4]. PTSD occurs in about 8% of pregnant women as reported in a study from the US [5], with the rate as high as 42% for displaced/migrating women [6]. Maternal exposure to stressful situations during pregnancy may have effects on their pregnancy, fetal development and birth outcomes which might result from the maternal endocrine and immune system response to these stressful stimuli [7, 8]. Research has found associations between adverse pregnancy and birth outcomes and higher risk of morbidity and mortality of the newborn, both in the short and long term [9–11]. However there are mixed results regarding the impact of stress-related illness in pregnancy and preterm or Low Birth Weight (LBW) [12–15]. Apart from these direct effects, the women experiencing stressful conditions during pregnancy are more likely to engage in poor health behaviours including smoking [16], alcohol and drug use [17], and inadequate care utilization [18], all of which may lead to adverse pregnancy outcomes.

LBW baby is defined by the international statistical classification of diseases as a live newborn with a birth weight less than 2500 g regardless of gestational age [19]. LBW mainly results from prematurity (birth before 37 completed weeks of gestation), or intrauterine growth restriction-IUGR (birth weight <  2.5 kg and gestational age > 37 weeks) or both. Reducing the rates of LBW is a major public health concern as it is estimated that around the world about 30 million babies are born with LBW each year and a quarter of them face severe short and long-term health consequences [20].

The northern part (districts of Swat, Dir and Buner) of Pakistan was a nexus of conflict and militancy between the Taliban and the security forces since July, 2008, resulting in a large scale displacement of the local population. Around 2 million had been registered to be displaced from the region. Only 20% of the displaced were living in 23 camps established by the Pakistan government, the rest were with relatives, rented accommodation, within family homes, in schools, mosques and other community buildings [21]. Most of the displaced people have targeted bigger cities of the province like, Peshawar, Nowshera and Mardan. It was during this context the present research was planned and conducted. The objective of the current research was to determine whether Post-Traumatic Stress Disorder (PTSD) is independently associated with increased risk of LBW baby. The conflict situation was a natural context to determine this association as most of the displaced persons were living in the major city of Peshawar.

## Methods

This case control study was conducted in three tertiary care hospitals of district Peshawar (Post Graduate Medical Institute Lady Reading Hospital, Khyber Teaching Hospital and Hayat Abad Medical Complex). These are tertiary level referral hospitals for the entire province and it was forecasted that most of the displaced people will avail services in these facilities. The total duration of the study was 4 months, from April 20th to July 3rd, 2009. The study population were single live newborns of 24 or more weeks of gestation and their mothers delivered at or admitted within 24 h of birth at the hospital maternity wards of the three tertiary care hospitals of Peshawar. Newborn with recognizable congenital anomalies, twin pregnancy, born to mothers whom labor was induced for medical complications or with maternal history of cervical incompetence were excluded. Mothers with known mental health problems or any other serious medical condition were excluded from the study. Cases were newborn mother pairs with the newborn weighing less than 2.5 kg delivered or admitted within 24 h of birth in the above mentioned hospital maternity wards. Similarly, controls were newborn mother pairs with the newborn weighing more than or equal to 2.5 kg delivered or admitted within 24 h of birth. Cases and controls were enrolled on 1:1 ratio. The controls were selected from the same maternity ward at the same time of the inclusion of the case by random sampling among the women with normal birth weight i.e. 2.5 kg or more.

To determine the study objective of finding the association between the LBW and the exposure to PTSD, the sample size was estimated based on the following assumptions: an alpha of 0.01, power of 90%, and assuming a prevalence of PTSD in the population at 10%, ratio of cases vs controls of 1:1, and an odds ratio of 3 as a measure of clinical significance. Using OpenEpi version 3.01 [22] a sample size of 404 was estimated i.e. 202 cases and 202 controls. Assuming a non-response rate of 10% for refusals, 450 sample size was determined with 225 cases and 225 controls.

### Instrument and procedures

#### MINI neuropsychiatric interview

For the diagnosis of current PTSD the MINI Neuropsychiatric Interview 5.0, a validated questionnaire that uses DSM-IV criteria was administered [23]. Studies have shown that the MINI Neuropsychiatric Interview 5.0 is a valid and reliable diagnostic tool used in epidemiological studies targeting pregnant women [24, 25]. The questionnaire was administered by trained medical officers in the respective hospitals. For a diagnosis of PTSD the subject was required to confirm experience of an “unusually traumatic or stressful event”, re-experiencing the event over the last month and at least 3 of the following: avoidance, amnesia, decreased interest in activities, detachment, numbing, foreshortened future, and at least 2 of the following: trouble sleeping, irritability, difficulty concentrating, nervousness, and feeling easily startled for at least “several days”. Lastly, the subject had to indicate whether the above problems had significantly interfered with the daily work or social activities or caused significant distress.

#### Birth weight

Birth weight of the newborn was measured by weighing the infant with minimum clothing on and recording the weight to the nearest 0.1 kg, by the trained medical officers. The standard cut-off for LBW in newborns was used i.e. 2.5 kg or less [26].

#### Maternal anthropometry and Anemia

Women’s height and weight measurements were recorded from the clinical records. Body Mass Index (BMI) calculations were later done during analysis. BMI was calculated using the standard as weight in kilogram divided by height in meter square classified according to WHO criteria as underweight (< 18.5), normal (18.5–24.9), overweight (25–29.9) and obese (> 30) [27]. Likewise, maternal anemia was measured from clinical records and was categorized as anemia with hemoglobin level less than 11 g/dl [28].

#### Socio-demographic characteristics

Data was also collected regarding the gender of the newborn, maternal age, maternal and paternal schooling, household income, rural/urban residence, Gravida, inter-pregnancy interval, and previous history of preterm births. Maternal age was participant reported at the time of interview. Maternal and paternal schooling were also recorded and was assessed through formal years of schooling and later categorized as less than 5 years and more than 5 years for analysis as the overall rates of literacy was very low in the study participants. Similarly, household income was categorized as less than or more than Rs. 10,000/month for analysis. Gravidity was defined as the total number of pregnancies including live births, still births and abortions and categorized as primigravida (first pregnancy), 2–3 Gravida (2–3 pregnancies) and 4 or more Gravida (≥4 pregnancies). Inter-pregnancy interval was defined as duration between conception for index pregnancy and the preceding delivery, abortion or stillbirth and was also self-reported by the mother.

The data collection was regularly supervised and monitored by the Principal Investigator (PI). The filled questionnaires by the data collection team were checked by the PI (HR) daily at the end of the day for their completeness. Data were coded and entered into the SPSS version 23 and analyzed using statistical package (STATA version 14). Descriptive analysis included frequencies and percentages for all the variables. For uni-variate analysis of the association between LBW and all other factors Chi square test (Fisher’s two-sided exact test was used if numbers in the cells < 5) was used. Associations were considered significant at 5%. Logistic regression analysis of the association between LBW and all those variables with a *p*-value of < 0.25 on uni-variate analysis were entered in the logistic regression model.

The study was approved by the ethical committee of Health Services Academy, Islamabad. Informed written consent obtained from study participants following explanation of the study aims and objectives. In case of illiterate women, the information was verbally explained to them in the local language and consent taken thereafter. The right to withdraw from the study was reinforced to all the participants throughout the study conduct. All information obtained in the study was kept confidential with access to personal information available only to the study lead (HR). Cases of severe distress and other health concerns requiring immediate treatment were referred to the concern department accordingly.

## Results

A total of 450 newborn and mother pairs participated in the study with 225 cases (LBW mother pairs) and 225 controls (normal weight babies and mother pairs). Table 1 shows the association of socio-demographic and other factors with LBW (cases) and normal weight babies (controls). Factors significantly associated with LBW include: less than 5 years of paternal schooling (*p* = 0.017) and PTSD (*p* <  0.001). All other factors were not found to be statistically significant and were; sex of the newborn child, maternal age, maternal schooling, monthly family income, rural/urban residence, Gravida, inter-pregnancy interval, previous history of preterm birth, maternal BMI and maternal anemia.

**Table 1:** Factors among cases and controls

Variables/Factors	Cases (LBW) ***N*** = 225 n (%)	Controls (NBW) ***N*** = 225 n (%)	P value
Male Neonate	131 (58.2)	126 (56.0)	0.634
Maternal Age			
20–35 yrs.	172 (76.4)	178 (79.1)	0.606
< 20 years	30 (13.3)	30 (13.3)	
> 35 years	23 (10.2)	17 (7.6)	
< 5 years of Maternal Schooling	150 (66.7)	163 (72.4)	0.183
< 5 years of Paternal Schooling	108 (48.0)	83 (36.9)	0.017
< 10, 000/Monthly Income (PKR)	157 (69.8)	167 (74.2)	0.294
Rural Residence	161 (71.6)	170 (75.6)	0.336
Primigravida	58 (25.8)	76 (33.8)	0.179
2–3 Gravida	67 (29.8)	60 (26.6)	
4 or more Gravida	100 (44.4)	89 (39.6)	
< 2 years Inter pregnancy Interval^a^	109 (65.3)	87 (58.4)	0.208
History of preterm birth	38 (16.9)	25 (11.1)	0.077
Normal (BMI 18.5–24.9)	181 (80.4)	183 (81.3)	0.746
Under weight (BMI < 18.5)	26 (11.6)	28 (12.4)	
Over weight (BMI 25-29.9)	18 (8)	14 (6.2)	
Maternal Anemia	111 (49.3)	107 (47.6)	0.706
PTSD	71 (31.6)	13 (5.8)	< 0.001

^a^primigravida excluded from analysis

Table 2 shows the uni-variate logistic regression analysis between the cases and controls and all the socio-demographic and other factors.. As is shown in the table 48% of the fathers of the cases had less than 5 years of schooling, compared to 36.9% of the controls; showing significant association with LBW: OR = 1.58 (95% CI 1.08 to 2.30). Around one third (31.6%) of the mother of the cases were diagnosed with PTSD, compared to only 5.8% of the controls: OR = 7.52 (95% CI 4.02 to 14.07). The association between LBW and all other variables was not found to be statistically significant.

**Table 2:** Factors and association with LBW among cases and controls

Variables/Factors	Cases (LBW) ***N*** = 225 n (%)	Controls (NBW) ***N*** = 225 n (%)	OR	95% CI	P value
Male Neonate	131 (58.2)	126 (56.0)	1.09	0.75 to 1.59	0.634
Maternal Age					
20–35 yrs.	172 (76.4)	178 (79.1)	Reference category		
< 20 years	30 (13.3)	30 (13.3)	1.03	0.60 to 1.79	0.902
> 35 years	23 (10.2)	17 (7.6)	1.40	0.72 to 2.71	0.318
< 5 years of Maternal Schooling	150 (66.7)	163 (72.4)	0.76	0.51 to 1.14	0.183
< 5 years of Paternal Schooling	108 (48.0)	83 (36.9)	1.58	1.08 to 2.30	0.017
< 10, 000/Monthly Income (PKR)	157 (69.8)	167 (74.2)	0.80	0.53 to 1.21	0.294
Rural Residence	161 (71.6)	170 (75.6)	0.81	0.53 to 1.24	0.336
Gravidity					
Primigravida	58 (25.8)	76 (33.8)	Reference category		
2–3 Gravida	67 (29.8)	60 (26.6)	1.46	0.90 to 2.38	0.126
4 or more Gravida	100 (44.4)	89 (39.6)	1.47	0.94 to 2.30	0.089
< 2 years Inter pregnancy Interval*	109 (65.3)	87 (58.4)	1.34	0.85 to 2.11	0.209
History of preterm birth	38 (16.9)	25 (11.1)	1.63	0.94 to 2.80	0.079
Maternal Body Mass Index					
Normal (BMI 18.5–24.9)	181 (80.4)	183 (81.3)	Reference category		
Under weight (BMI < 18.5)	26 (11.6)	28 (12.4)	0.94	0.53 to 1.66	0.829
Over weight (BMI 25-29.9)	18 (8)	14 (6.2)	1.30	0.63 to 2.69	0.480
Maternal Anemia	111 (49.3)	107 (47.6)	1.07	0.74 to 1.55	0.706
PTSD	71 (31.6)	13 (5.8)	7.52	4.02 to 14.07	< 0.001

To study the association of combined effects of all these variables on the risk of being a LBW, a logistic regression model was created. In this model, only those variables that had a *p* value of < 0.25 in the uni-variate regression model were included in the analysis. These include maternal education, paternal education, Gravida status, inter-pregnancy interval, history of preterm birth and PTSD. The logistic regression model (Table 3) found that PTSD was significantly associated with LBW in the presence of other variables i.e. maternal education, paternal education, Gravida status, inter-pregnancy interval and history of preterm birth. Mothers of LBW babies were 7.52 times more likely to have been exposed to PTSD independent of other risk factors/variables.

**Table 3:** Logistic Regression Analysis

Variables/Factors	Unadjusted OR	95% CI	Adjusted OR	95% CI
Male Neonate	1.09	0.75 to 1.59	–	–
Maternal Age				
20–35 yrs	Reference category			
< 20 years	1.03	0.60 to 1.79	–	–
> 35 years	1.40	0.72 to 2.71	–	–
< 5 years of Maternal Schooling	0.76	0.51 to 1.14	0.41	0.23 to 0.75
< 5 years of Paternal Schooling	1.58	1.08 to 2.30	1.39	0.81 to 2.38
< 10, 000/Monthly Income (PKR)	0.80	0.53 to 1.21	–	–
Rural Residence	0.81	0.53 to 1.24	–	–
Primigravida	Reference category			
2–3 Gravida	1.46	0.90 to 2.38	–	–
4 or more Gravida	1.47	0.94 to 2.30	1.05	0.64 to 1.73
< 2 years Inter pregnancy Interval*	1.34	0.85 to 2.11	1.55	0.94 to 2.56
History of preterm birth	1.63	0.94 to 2.80	1.41	0.77 to 2.58
Normal (BMI 18.5–24.9)	Reference category			
Under weight (BMI < 18.5)	0.94	0.53 to 1.66	–	–
Over weight (BMI 25-29.9)	1.30	0.63 to 2.69	–	–
Maternal Anemia	1.07	0.74 to 1.55	–	–
PTSD	7.52	4.02 to 14.07	6.07	2.99 to 12.31

## Discussion

This study found that PTSD exposure during pregnancy is independently associated with the risk of LBW in the presence of other risk factors like maternal education, paternal education, Gravida status, inter-pregnancy interval and history of preterm birth. The study was conducted in a context where active conflict was ongoing in the northern part of Khyber Pakhtunkhwa and the population was displaced to the major cities. Therefore, the current study was quite unique as it found association in real world with active conflict affected participants.

The key finding of the current study was that PTSD was independently associated with LBW. Similar results have been shown in literature from other areas with conflict or some other terror attacks or generalized stress or distress. In a prospective cohort study conducted in Sau Palo Brazil, 865 pregnant women who attended antenatal care between September 1997 and August 2000 were assessed for stress and distress by interview during the three trimesters of pregnancy. Maternal distress was associated with LBW in the second trimester and with prematurity in the third trimester in the presence of other risk factors. The prevalence of stress and distress in the different trimesters of pregnancy varied from 22.1 to 52.9% [29]. Similarly a cohort study by Seng et al. identified PTSD as a strong predictor of poor birth outcome in terms of LBW and prematurity [30]. Similarly another cohort study showed same results, as women with PTSD (PTSD assessed with MINI neuropsychiatric interview as in the current study) were at greater risk of delivering preterm babies compared those who were not diagnosed with PTSD [14]. The results were not statistically significant as the cohort had very small numbers of PTSD and hence the sample was underpowered. On the other hand, minor depressive disorder was significantly associated with LBW [14].

Literature has found some contrasting results as well. A study was conducted in USA to see the effect of extreme trauma on the birth outcomes of women highly exposed to the World Trade Center on 11 September 2001 [13]. The study did not found any significant association between the terror attack and adverse perinatal outcomes. The difference was that they enrolled women mostly in their first trimester on 11^th^September and the sample size was small as compared to the current study. Apart from that the event was a onetime exposure and was an acute one. While in the current study it was a chronic exposure to the traumatic and terror acts and these women were displaced from their houses and were living in camps, with relatives or rented accommodation. Research has indicated that persistent exposure to stressful stimuli for long periods make the perinatal outcomes worse. A further explanation could be that most of their sample was in the first trimester which as discussed in the other study [29] above did not have any association with poor pregnancy outcome.

Maternal age and association with LBW was not found to be statistically significant when compared for mothers below 20 and above 35 years of age. Similar findings were observed in a case control study in the present context where no association was observed between LBW and teenage pregnancy [31]. However, a cohort study revealed higher risk of preterm and LBW in adolescent (< 18 years) mothers [32]. Furthermore, inter-pregnancy interval has been shown to be associated with LBW [33, 34]. In the present study again no such association was observed possibly as we excluded primigravida from this analysis and the remaining sample may be under powered, although the odds were more for the LBW mothers. Low pre-pregnancy Body Mass Index (BMI) is one of the strongest predictors of adverse pregnancy outcomes such as preterm birth and fetal growth retardation. BMI of < 18.5 was found to be significantly associated with LBW in a study [35]. Similarly, mothers who were underweight, were 3.4 times more likely to give birth to an IUGR baby [36]. However, in the current study BMI (both high and low) was not found to be statistically associated with LBW. The main reason could be that these measures were not recorded for the pre-pregnancy time and were only available at the time of data collection during the hospital.

Others factors found to be associated with LBW, as reported by previous studies, include pregnancy related complications, acute/chronic infections during pregnancy [37], and lack of antenatal care [37, 38]. Similarly exposure to other stressful events during pregnancy such as natural disasters (earthquakes, hurricane) [39], childhood maltreatment history [30], domestic violence, food insufficiency [40], were also identified as risk factors for LBW. However, investigating those factors was beyond the scope of the current study.

The major strength of the current study was that the data collection was done at a time when most of the districts were exposed to conflict and militancy in the area. Large number of people migrated and were living in camps, with relatives or rented accommodation in district Peshawar. Therefore, the study captured the real occurrence of PTSD and its association with LBW. As data was collected from the three main tertiary care facilities therefore could be generalized with caution. Moreover, the tool used for the main exposure variable of PTSD (MINI Neuropsychiatric Interview 5.0) is a valid and reliable diagnostic tool with high inter-rater and test–retest reliabilities and is considered as a gold standard for psychiatric disorders [25]. The main limitation of the current study was that we used women records for some of the factors like anemia and pre pregnancy BMI, the authenticity of these variables could not be guaranteed. Moreover Genetic, behavioral and environmental factors might be associated with LBW and were not assessed in the current study. Furthermore, a prospective cohort study would be a better design to investigate the current association.

## Conclusions

To conclude PTSD was found to be independently associated with LBW in the displaced population following the conflict in the Malakand division of Pakistan. LBW is a major public health problem as most of the children face short and long-term consequences [20], therefore intervention programs should be considered for pregnant women exposed to traumatic events and further studies are recommended in the local context to evaluate the effectiveness of such interventions. Based on the current study we can infer that stresses during antenatal period negatively influence fetal growth. However, other factors including genetic, behavioral and environmental factors need to be explored.

## Data Availability

The raw data available upon reasonable request from the corresponding author.

## Abbreviations

BMI: Body mass index
CI: Confidence interval
DSM: Diagnostic and statistical manual
EMR: Eastern Mediterranean Region
IDPs: Internally displaced persons
IUGR: Intrauterine growth restriction
Kg: Kilogram
LBW: Low birth weight
OR: Odds ratio
PTSD: Post-traumatic stress disorder
USA: United States of America
WHO: World Health Organization
